# Changes in NMDA receptor contribution to synaptic transmission in the brain in a rat model of glaucoma

**DOI:** 10.1016/j.nbd.2010.04.019

**Published:** 2010-09

**Authors:** A.L. Georgiou, L. Guo, M.F. Cordeiro, T.E. Salt

**Affiliations:** Department of Visual Neuroscience, UCL Institute of Ophthalmology, 11-43 Bath Street, London EC1V 9EL, UK

**Keywords:** Superior colliculus, Glaucoma, Retinal degeneration, Retinal ganglion cell, NMDA receptors, Glutamate, Synaptic transmission, Rodent visual system

## Abstract

In the age-related, blinding disease glaucoma, retinal ganglion cells (RGCs) degenerate, possibly affecting glutamatergic retinofugal transmission to the brain. The superior colliculus (SC) is a major central target of retinofugal axons in the rodent, a much used disease model. We investigated the contribution of NMDA-type glutamate receptors to retinocollicular transmission in a rat glaucoma model, using a SC brain slice preparation to determine the sensitivity of synaptic responses to the NMDAR antagonist D-AP5. At 32 weeks after induction of experimental glaucoma, but not earlier, there was an increase in NMDAR contribution to SC synaptic responses in slices receiving input from glaucomatous eyes. This suggests that there are changes in NMDAR function after RGC degeneration in experimental glaucoma, which may represent functional SC compensation through plasticity via NMDARs. This has implications for studies carried out using rodent glaucoma models, especially those evaluating potential treatment strategies, as it suggests that functional changes in the central visual system need to be considered in addition to those in the eye. Furthermore, the data underline the need for early therapeutic intervention in order to pre-empt subsequent central functional changes.

## Introduction

Glaucoma is one of the leading causes of blindness worldwide (Resnikoff et al., 2004). The disease leads to the apoptosis and degeneration of the neurones that transfer visual information to the central visual areas, the retinal ganglion cells (RGCs). Previous studies in glaucomatous patients (Kiyosawa et al., 1989; Miki et al., 1996; Sugiyama et al., 2006; Duncan et al., 2007a,b) and in experimental glaucoma models in primates (Imamura et al., 2009) have shown that the central visual areas also show functional changes at the cortical and sub-cortical level (Smith et al., 1993). Furthermore, anatomical changes have also been seen in the lateral geniculate nucleus, where RGC axons terminate, in a primate model of glaucoma (Yucel et al., 2006). Thus, there is increasing evidence that glaucoma is a condition that has consequences for visual areas in the brain.

Many studies looking at disease mechanisms and potential treatment strategies for glaucoma are initially carried out using rodent models, and there is extensive information available on the progression of retinal degeneration in rodent glaucoma (Morrison, 2005; Pang and Clark, 2007). By contrast, relatively little is known concerning changes in central target areas of retinal axons in rodent glaucoma models. In rodents, the major central visual system contact for RGCs is the superior colliculus (SC) however there are few studies looking at the function of this area in a rat model of glaucoma. The study by King et al. (2006) found, using extracellular multiunit recordings that there was an expansion of the visual receptive fields of cells in the superior colliculus in a rat glaucoma model with elevated intraocular pressure. This result was suggested to indicate a possibility for plasticity and compensation in the retina and superior colliculus after RGC degeneration in this model. However these mechanisms to date have not been investigated. Furthermore, using an approach with visual stimulation makes it difficult to distinguish between effects that are due directly to retinal dysfunction and those effects that are genuinely central.

Glutamate is the major excitatory neurotransmitter in the visual pathway from the RGCs to the superior colliculus with NMDA receptors being found postsynaptically (Mize and Butler, 1996; Jeon et al., 1997). NMDA receptors have been shown to be important for the development and refinement of the correct topographic projections from RGCs to the superior colliculus (Simon et al., 1992; Shi et al., 1997). Previous studies from our laboratory have also shown the importance for NMDA receptors in visual function of the rat superior colliculus during development and in adulthood (Roberts et al., 1991; Binns and Salt, 1994, 1998). In addition to the glutamatergic excitatory processes, there are substantial inhibitory influences within the superior colliculus with all three types of GABA receptor (GABA_A_, GABA_B_, and GABA_C_) being seen in the area from birth (Bowery et al., 1987; Chu et al., 1990; Boue-Grabot et al., 1998; Wegelius et al., 1998; Pasternack et al., 1999; Clark et al., 2001). Around 45% of the cell population within the visual layers of the superior colliculus have been shown to be GABA-ergic neurones (Mize, 1988). All three groups of GABA receptor have been shown to play a role in visual processing in the superior colliculus (Arakawa and Okada, 1988; Hirai et al., 1993; Binns and Salt, 1997; Boller and Schmidt, 2001, 2003; Schmidt et al., 2001) with indications of their role in processes such as response habituation (Binns and Salt, 1997; Platt and Withington, 1997) long term depression (Mize and Salt, 2004) and long term potentiation (Platt and Withington, 1998; White and Platt, 2000, 2001).

As both NMDA and GABA receptors have been shown to have an important role in visual processing in the superior colliculus it is likely that changes that may occur to these receptors due to degeneration of the RGC input may affect the ability of the area to function correctly. Thus we have investigated the changes that may occur in the contribution of these receptors to synaptic transmission in the superior colliculus in a rat ocular hypertension (OHT) model of glaucoma. We chose an *in vitro* slice preparation of the superior colliculus so as to eliminate the complications that are involved when using *in vivo* techniques with visual stimulation. In our preparation, optic tract stimulation elicits a field post-synaptic potential (fPSP) in the superficial grey layer of the superior colliculus that has NMDA receptor and GABA receptor contributions that can be revealed by application of NMDA receptor and GABA receptor antagonists. Using such an approach, we investigated the timeline of any changes in the contribution of these receptors to the fPSP in the superior colliculus at 3, 16 and 32 weeks after OHT induction, and we found that there was an increase in NMDA receptor contribution to synaptic responses at the final time point.

## Methods

All conditions and experimental procedures were carried out in accordance with the UK Animals (Scientific Procedures) Act 1986 and associated guidelines. Animals were obtained from Harlan UK and given at least 48 hours to accommodate before any procedures were performed. 44 male Dark Agouti rats age 7–8 weeks (150–200 g) were housed in a 12 hour light/12 hour dark cycle with unlimited access to food and water. 18 of the animals had the ocular hypertension (OHT) model induction performed with the other 26 being used as age matched unoperated controls.

For induction of the OHT model of glaucoma, animals were anesthetised using an intraperitoneal injection of a ketamine (100 mg/ml) (37.5%)/medetomidine (1 mg/ml) (25%)/sterile water (37.5%) solution at 0.2 ml/100 g. Unilateral elevation of intraocular pressure (IOP) was induced in the left eye only of 18 animals using an adaptation of the Morrison method (Morrison et al., 1997) which has been described, characterised and validated in our lab (Cordeiro et al., 2004; Guo et al., 2005a,b, 2006, 2007). Briefly, IOP was elevated by injection of 50 μl of hypertonic saline solution (1.8 M) into the episcleral veins, using a syringe pump (60 µl/min; UMP2, World Precision Instruments, Sarasota, FL). Antibiotic ointment was then applied to the eye. Animals were then allowed to recover from anaesthesia.

Baseline IOP measurements were made before IOP elevation, they were then also measured after OHT induction at weekly intervals for a month and monthly intervals thereafter using a rebound tonometer (custom built at Department of Ophthalmology, Mount Sinai School of Medicine, New York, NY) while animals were under isoflurane anaesthesia (Merial; Animal Health, Ltd., Essex, UK). The differences between IOP values of the unoperated and the IOP elevated eyes were measured and statistical analysis was then performed using a one-way ANOVA and Tukey honestly significant difference (HSD) post hoc analysis using SPSS.

For the superior colliculus *in vitro* slice preparation animals from the OHT model group and the age matched control group were taken at either 3, 16 or 32 weeks after IOP elevation or unoperated control at the corresponding ages. Animals were anesthetised as above and left for 30 min. Animals were then decapitated and their brains were removed rapidly and placed into ice-cold, oxygenated Krebs medium containing (mM): sucrose 202, KCl 2, KH_2_PO_4_ 1.25, MgSO_4_ 10, CaCl_2_ 0.5, NaHCO_3_ 26, and glucose 10. The cerebellum and a small amount of frontal cortex were removed and cuts were made on the right and left hand sides parallel to the midline so that a flat surface was revealed. The flat surface was then glued on one side to the cutting stage of a vibratome (Integraslice 7550 MM, Campden Instruments, Lafayette, Indiana, USA) with cyanoacrylate adhesive and parasagital slices of 400 μm thickness were cut through the superior colliculus. This method allows the optic tract and its connections to the superficial layers of the superior colliculus to be maintained in slices from both sides of the brain. Given that the retinal projection to the SC is more than 90% crossed (Dreher et al., 1985), recordings were possible from SC slices with RGC input from both the operated and unoperated eyes from the same animal. After cutting, slices were transferred to a bath containing oxygenated Krebs medium containing (mM): NaCl 124, KCl 2, KH_2_PO_4_ 1.25, MgSO_4_ 5, CaCl_2_ 1, NaHCO_3_ 26, and glucose 10. Slices were then left for at least 1 h before being transferred to an interface recording chamber where they were perfused at a rate of 0.7 ml/min with warmed (33–34 °C) oxygenated Krebs medium containing (mM): NaCl 124, KCl 2, KH_2_PO_4_ 1.25, MgSO_4_ 1, CaCl_2_ 2, NaHCO_3_ 26, and glucose 10.

Stimulation to the optic tract immediately prior to its entry to the SC was via a bipolar tungsten-in-glass electrode with extracellular recordings being made in the superficial grey layer of the SC via a Krebs-filled glass micropipette (5–10 μm in diameter). Responses were recorded using an Axoprobe 1A amplifier (Axon Instruments), digitised at 10 kHz via a CED1401 interface and stored on a computer using Spike 2 software (Cambridge Electronic Design).

Each recording session was started by determining a minimum and maximum response to stimulus intensity (0.1 ms pulses, 20 s interval) for the slice and the intensity level was then set at 75% of that which produced the maximum response. A stable baseline was then recorded for 20 min. The GABA antagonists Picrotoxin (100 μM, Sigma-Aldrich) and CGP55845 (3 μM, Tocris) were then added together to the bathing medium for at least 20 min. This combination of antagonists is known to block all three types of GABA receptor (Bormann, 2000). The NMDA receptor antagonist D-AP5 (100 μM, Ascent Scientific) was then added with the GABA antagonists to the bathing medium for 10 min.

**Fig. 1:**
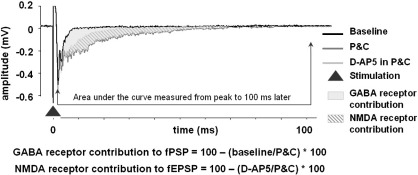
Measurements for area under the curve for the late phase of the fEPSP and calculations for receptor contribution. The figure shows how the measurements of area under the curve were taken and the calculations for both GABA receptor contribution and NMDA receptor contribution to the fEPSP. The area under the curve for each of the conditions was measured from the peak of the fEPSP to 100 ms after this. The contribution of the GABA receptors to the fEPSP was measured using the calculation shown and is seen in grey shading in the figure. Application of the NMDA receptor antagonist (D-AP5) reduced the amplitude and latency of the late phase of the excitatory fEPSP (revealed when GABA antagonists were added) and the contribution of these receptors was measured using the calculation shown and is seen in the figure in grey hashing.

Responses to stimuli were waveform averaged (3 trials, equal to 60 s of recording) and the peak amplitude of the fEPSP was measured. In addition, area under the curve measurements were made from the peak of the fEPSP to 100 ms after stimulation to assess the effect of GABA and NMDA antagonists on the late phase of the fEPSP (Fig. 1). The fEPSP amplitude measurements taken for the last 3 min of drug application were normalised and compared to baseline condition measurements. In addition, the latency to peak of the fEPSP and the duration of the fEPSP were measured at the same time points. The effects of drugs on all these fEPSP parameters were statistically analysed using paired *t*-tests and differences between the groups for amplitude and receptor contribution measurements were analysed using one-way ANOVA and a post hoc Tukey HSD test using SPSS software.

## Results

**Fig. 2:**
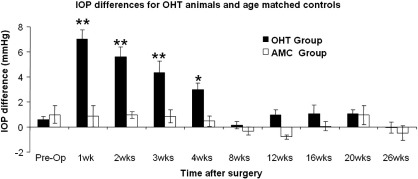
IOP differences in left (IOP elevated) and right (unoperated) eyes in OHT model and age matched unoperated control (AMC) animals. The figure shows that there was a significant elevation of IOP in the operated eye compared to the unoperated eye for at least 4 weeks in OHT animals. In age matched controls there were no significant differences in IOP values at any of the time points measured. Values indicate IOP difference between operated and unoperated eyes with error bars indicating standard error of mean (** *P* < 0.01; * *P* < 0.05).

Measurements of IOP in the OHT model animals between 1 week and 4 weeks after IOP elevation showed that there was a significantly greater IOP in the IOP elevated eyes compared to the unoperated eyes (Fig. 2). This returned to baseline levels by 8 weeks after IOP elevation in a manner similar to that seen previously in our laboratory (Guo et al., 2006, 2007). In unoperated control animals there were no significant differences in the IOP of the left or right eyes at any of the time points measured.

Stimulation of the optic tract in our parasagital SC slices typically evoked fEPSPs in the superficial grey matter similar to those that we have described previously in adult rats (Cirone et al., 2002; Pothecary et al., 2005). Responses to stimulation under control conditions in unoperated animals had a peak amplitude of around −0.7 mV and latency to peak of 2 ms, and similar results were obtained irrespective of from which side of the brain slices had been obtained (Tables 1 and 2).

### GABA receptor contribution to synaptic responses

**Fig. 3:**
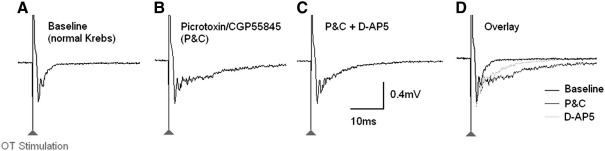
The effect of addition of GABA antagonists (Picrotoxin and CGP55845) and NMDA receptor antagonist (D-AP5) on the fEPSP in a slice taken from an age matched unoperated control animal aged 22 weeks. A - fEPSP recorded under control conditions in normal Krebs solution. B - When GABA antagonists to all 3 types of GABA receptor were applied this revealed an enhanced excitatory component of the fEPSP. C - When the NMDA receptor antagonist D-AP5 (100 μM) was added in addition to the GABA antagonists this caused a reduction in the late phase of the fEPSP, indicating the contribution of the NMDA receptors to this excitatory component of the response. D - Overlay of traces *A*,*B* & *C*.

Application of the combination of GABA antagonists revealed an increased amplitude and duration of the late phase of the fEPSP (Fig. 3) as seen in previous studies from our laboratory (Mize and Salt, 2004; Pothecary et al., 2005). In unoperated age-matched controls there was no significant difference in the contribution of GABA receptors to the synaptic response in slices from the left superior colliculus compared to slices from the right superior colliculus at 10 weeks old, 23 weeks old, or 39 weeks old, corresponding to 3, 16, and 32 weeks after the OHT elevation (Table 3). The duration of the late phase of the fEPSP while GABA receptor antagonists were applied was not significantly different in slices from the left or right superior colliculus at any of the time points (Table 4).

**Fig. 4:**
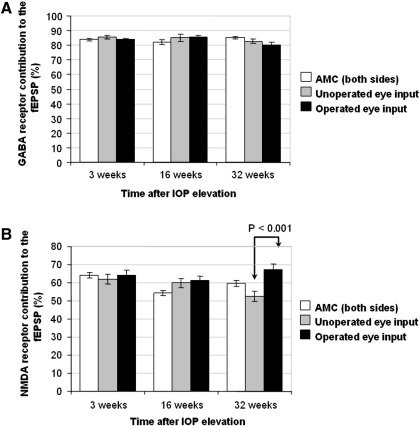
GABA receptor (A) and NMDA receptor (B) contribution to the fEPSP in OHT model animals. A -There were no significant differences in the contribution of GABA receptors to the fEPSP between slices with input from the glaucomatous eyes compared to those with input from the unoperated eyes in OHT model animals at any of the time points tested. Age matched control (AMC) data from slices from both hemispheres of the superior colliculus are shown for comparison in both graphs. B -There were no significant differences in the NMDA receptor contribution to the excitatory fEPSP between slices with input from the glaucomatous eyes compared to those with input from the unoperated eyes at 3 or 16 weeks after IOP elevation. However at 32 weeks after IOP elevation there was a significantly greater contribution of NMDA receptors to the excitatory fEPSP in slices with input from the glaucomatous eyes compared to those with input from unoperated eyes.

In animals where OHT glaucoma had been induced, the contribution of the GABA receptors to the synaptic response was also not significantly different in superior colliculus slices receiving input from the unoperated eyes compared with those with input from the glaucomatous eyes at 3, 16 or 32 weeks after IOP elevation (Table 3, Fig. 4 A). The duration of the late phase of the fEPSP while GABA receptor antagonists were applied was not significantly different in superior colliculus slices with input from the unoperated eyes or glaucomatous eyes at any of the time points (Table 5).

In both age matched control animals and OHT animals, at all time points, the addition of GABA antagonists caused an increase in the peak amplitude of the fEPSP although this was not always significant (Table 1). There were no significant changes in the latency of the peak of the fEPSP due to addition of GABA receptor antagonists in age matched controls or OHT animals at any of the time points (Table 2).

### NMDA receptor contribution to fEPSPs

Removal of GABAergic inhibition by application of GABA antagonists revealed a fEPSP that is a composite of non-NMDA receptor and NMDA receptor-mediated components (Cirone et al., 2002; Pothecary et al., 2005). Under these conditions it was possible to investigate the relative contribution of NMDA receptors and non-NMDA receptors to fEPSPs by applying an NMDA antagonist in addition to the GABA antagonist combination. Thus, application of the NMDA receptor antagonist D-AP5 reduced the amplitude and duration of the late phase of the fEPSP (Fig. 3) as has been previously shown in our laboratory (Pothecary et al., 2005). In unoperated age matched control animals there was no significant difference in the contribution of the NMDA receptors to the excitatory fEPSP between the left and right superior colliculus slices in any of the age groups (Table 3). Furthermore, the duration of the late phase of the excitatory fEPSP while D-AP5 was applied was not significantly different in left or right superior colliculus slices at any of the time points (Table 4), indicating that there were no differences in the non-NMDA receptor mediated component of the fEPSP in these animal groups.

**Fig. 5:**
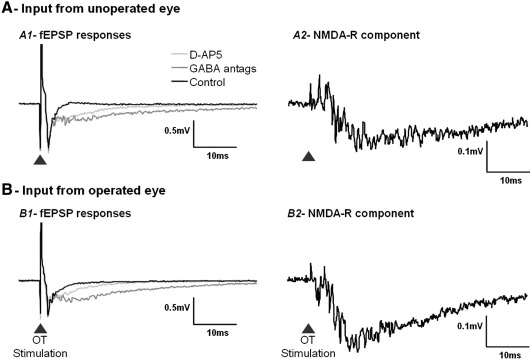
Examples of the effect of addition of GABA antagonists (Picrotoxin and CGP55845) and NMDA receptor antagonist (D-AP5) on the fEPSP in slices with input from the operated eye and those with input from the unoperated eye at 32 weeks after IOP elevation (left-hand side overlaid traces). The contribution of NMDA receptors to these fEPSPs is shown as the difference between the GABA antagonist traces and D-AP5 traces on the right-hand side of each row. A - An example from a slice with input from the unoperated eye showing the contribution of NMDA receptors to the fEPSP. In this slice there was an 85% contribution of the GABA receptors to the response and 54% contribution of the NMDA receptors to the fEPSP (see Fig. 1 for details of calculation). B - An example from a slice with input from the operated (IOP elevated) eye, showing a greater effect of the NMDA receptor antagonist (D-AP5) on the fEPSP. In this slice there was an 80% contribution of the GABA receptors to the response and 68% contribution of the NMDA receptors to the fEPSP.

The contribution of the NMDA receptors to the fEPSP was not significantly different in superior colliculus slices receiving input from the unoperated eyes compared to those with input from the glaucomatous eyes at 3 weeks or 16 weeks after IOP elevation (Table 3). However at 32 weeks after IOP elevation there was a significant increase in the NMDA receptor contribution to the excitatory fEPSP in slices receiving input from the glaucomatous eyes (67 ± 3%) compared to slices receiving input from the unoperated eyes (53 ± 2.9%, *P* = 0.001, Fig. 4 B) and to slices from the age matched unoperated control animals (60 ± 1.6%, *P* = 0.046). Slices with input from the unoperated eyes in the OHT model animals did not show any significant difference in NMDA receptor contribution to the fEPSP compared to the age matched unoperated control slices (*P* = 0.083). Examples of the traces seen for the fEPSPs in slices with input from the IOP elevated (operated) eyes compared to those with input from the unoperated eyes at 32 weeks after IOP elevation in OHT model animals can be seen in Fig. 5.

The duration of the late phase of the fEPSP while D-AP5 was applied was not significantly different in superior colliculus slices with input from the unoperated eyes or glaucomatous eyes at any of the time points after OHT induction (Table 5), indicating that there was no difference in the non-NMDA synaptic component between these groups of animals.

Application of the NMDA antagonist D-AP5 caused no significant change in the peak amplitude of the fEPSP in age matched control animals and in the OHT animals it either caused a small decrease or no significant change (Table 1). There was no significant effect of D-AP5 application on the latency of the peak of the fEPSP in age matched unoperated controls or OHT model animals (Table 2).

## Discussion

In the present study we have found that the NMDA receptor contribution to the optic tract-evoked fEPSP was significantly increased in superior colliculus slices with input from the glaucomatous eyes compared to those with input from the unoperated eyes. Interestingly, these changes were seen at 32 weeks after induction of OHT glaucoma rather than at the earlier time points after IOP elevation. Thus the NMDA receptor contribution to synaptic transmission in the superior colliculus is changed in this rat OHT model of glaucoma only at the later stage of the disease and this may be due to changes in the expression and/or function of these receptors. A previous study in an enucleation model of RGC degeneration showed no change in the expression of NMDA receptors in the superior colliculus up to 20 days after surgery (Chalmers and McCulloch, 1991b). However, and perhaps more relevant, a study in a primate model of glaucoma has shown that the NR1 subunit of the NMDA receptor is increased in the LGN layers connected to the glaucomatous eye 8-11 months after IOP elevation but not earlier (Yucel et al., 2006). This data by Yucel et al. (2006) showing an increase in the NR1 subunit suggests the speculation that a similar change is occurring in the superior colliculus in our rat model of glaucoma, indicating that the changes in NMDA receptor contribution seen in the present study may be due to increased NMDA receptor expression. However, this does not exclude other possibilities such as dendritic changes that may result in differences in synaptic transmission (Gupta et al., 2007; Liu et al., 2008).

Previous work from this laboratory has shown that in this OHT glaucoma model, the apoptosis rate of RGCs peaks at ∼ 3 weeks after OHT induction, but that there is a continuing increasing loss of RGCs, so that by 16 weeks after IOP elevation the loss of RGCs exceeds 50% (Cordeiro et al., 2004). This indicates that few central changes occur during the early stages of the degeneration when apoptosis is maximal but when the total RGC *loss* is still relatively little. Our finding that it is only later than 16 weeks that there is a significant change in NMDA receptor contribution to SC responses suggests that it is not apoptosis as such, but a substantial and relatively prolonged RGC degeneration that is required to provoke central changes. This would be consistent with the NMDA receptor expression studies in primate glaucoma models, showing elevated NR1 expression at later stages of the disease (Yucel et al., 2006). Our findings thus suggest that if the progression of the disease could be halted at an early stage (*e.g*. by reduction in peak apoptosis) then central changes may be prevented. This indicates that early therapeutic intervention in glaucoma is not only desirable for retaining retinal function, but also for retaining normal function in retino-recipient brain areas.

Our finding in that a large contribution of NMDA receptors to the optic-tract evoked fEPSP is revealed when GABAergic inhibition is blocked is consistent with our previous findings (Pothecary et al., 2005). Similarly, Pothecary et al. (2005) found that in the later stages of retinal degeneration in the Royal College of Surgeons (RCS) dystrophic rat, there is an increased NMDA receptor contribution to SC synaptic responses. This suggests that there may be common central consequences of retinal degeneration or RGC degeneration in different disease syndromes and that this includes changes in NMDA receptor contribution to synaptic transmission. Furthermore, given the importance of NMDA receptors in synaptic plasticity and learning, both during development and in adulthood (Malenka and Nicoll, 1993; Collingridge and Bliss, 1995; Constantine-Paton, 2000; Rao and Finkbeiner, 2007; Citri and Malenka, 2008; Cohen and Greenberg, 2008), an increase in NMDA receptor function in the retinal disease syndromes suggests that there may also be scope for increased synaptic plasticity in these conditions. This suggests that there is possibility for plasticity within the superior colliculus after RGC degeneration and the loss of retinal input to the area which may hold promise for recovering visual function.

The present study has also confirmed the large inhibitory influence of GABA receptors on synaptic transmission in the superior colliculus as seen in previous studies by our lab (Cirone et al., 2002; Pothecary et al., 2002; Mize and Salt, 2004; Lacey et al., 2005; Pothecary et al., 2005). Previous studies have shown that up to 20 days after eye enucleation there is no change in the GABA_A_ receptor expression in the contralateral superior colliculus (Segu et al., 1986; Chalmers and McCulloch, 1991a). The data from the present study showing no change in the GABA receptor contribution to the SC synaptic response in the glaucoma model appears to agree with this data. However, although the overall contribution of all 3 receptor types appears to be unchanged this does not mean that there are no changes in the GABA receptors at all, and it may be that there are more subtle specific changes in progress. For example, other studies in eye enucleation models have suggested that the GABA-ergic neurons and terminals within the contralateral superior colliculus may show some rearrangement 6 weeks after surgery (Lund and Lund, 1971; Houser et al., 1983), and Turner et al. (2005) found changes in SC inhibitory circuitry following optic nerve transection. Therefore it would be interesting in future studies to look at the expression of individual GABA receptor types in the superior colliculus in rat glaucoma.

The data from both the present and the previous studies discussed suggest the importance for further studies into the changes that occur in the central areas consequent to RGC degeneration. These changes will need to be considered when designing treatments for retinal degenerative diseases including glaucoma.

## Conclusions

The data from this study show that there are functional changes that occur in the central visual targets for RGCs in rodent glaucoma models. In particular, we have seen that there is an increase in the contribution of NMDA receptors to synaptic transmission in the superior colliculus in the rodent OHT glaucoma model, and that these changes occur at a relatively late stage after the main phase of apoptotic RGC loss. The data suggest firstly, that early therapeutic intervention in glaucoma may be important to prevent CNS changes, and secondly that there may be a potential ability for central areas to show plasticity following the loss of retinal input, and this may aid in the design of future treatment for limiting visual loss in glaucoma.

## Figures and Tables

**Table 1:** The effect of GABA and NMDA receptor antagonists on peak amplitude of the fEPSP in age matched controls and OHT model animals. Values are average (mV) ± SEM of *n* values.

	Baseline (normal Krebs)	P&C	*P*-value	Baseline (P&C)	DAP5	*P*-value
A - 3 weeks after IOP elevation						
Age matched controls: Left (*n* = 9)	−0.71 ± 0.08	−0.8 ± 0.09	*P* = 0.013	−0.79 ± 0.08	−0.79 ± 0.09	*P* = 0.782
Age matched controls: Right (*n* = 8)	−0.67 ± 0.08	−0.72 ± 0.09	*P* = 0.013	−0.7 ± 0.08	−0.69 ± 0.07	*P* = 0.492
OHT: Left (input from unoperated eye) (*n* = 10)	−0.54 ± 0.02	−0.55 ± 0.03	*P* = 0.618	−0.55 ± 0.03	−0.52 ± 0.03	*P* = 0.001
OHT: Right (input from operated eye) (*n* = 10)	−0.57 ± 0.04	−0.54 ± 0.06	*P* = 0.265	−0.54 ± 0.06	−0.52 ± 0.06	*P* = 0.269
B - 16 weeks after IOP elevation						
Age matched controls: Left (*n* = 17)	−0.7 ± 0.05	−0.78 ± 0.07	*P* = 0.000	−0.77 ± 0.07	−0.8 ± 0.07	*P* = 0.271
Age matched controls: Right (*n* = 14)	−0.56 ± 0.03	−0.6 ± 0.05	*P* = 0.119	−0.6 ± 0.05	−0.61 ± 0.06	*P* = 0.095
OHT: Left (input from unoperated eye) (*n* = 10)	−0.46 ± 0.04	−0.47 ± 0.05	*P* = 0.655	−0.47 ± 0.05	−0.45 ± 0.05	*P* = 0.042
OHT: Right (input from operated eye) (*n* = 10)	−0.52 ± 0.05	−0.54 ± 0.08	*P* = 0.700	−0.54 ± 0.07	−0.55 ± 0.08	*P* = 0.597
C - 32 weeks after IOP elevation						
Age matched controls: Left (*n* = 15)	−0.54 ± 0.03	−0.57 ± 0.04	*P* = 0.106	−0.57 ± 0.04	−0.55 ± 0.04	*P* = 0.372
Age matched controls: Right (*n* = 13)	−0.62 ± 0.03	−0.69 ± 0.04	*P* = 0.002	−0.69 ± 0.04	−0.69 ± 0.04	*P* = 0.829
OHT: Left (input from unoperated eye) (*n* = 12)	−0.62 ± 0.03	−0.72 ± 0.05	*P* = 0.003	−0.71 ± 0.05	−0.73 ± 0.06	*P* = 0.171
OHT: Right (input from operated eye) (*n* = 14)	−0.54 ± 0.03	−0.56 ± 0.03	*P* = 0.123	−0.56 ± 0.03	−0.55 ± 0.04	*P* = 0.028

**Table 2:** The effect of GABA and NMDA receptor antagonists on latency of the peak of the fEPSP in age matched controls and OHT model animals. Values are average (ms) ± SEM of *n* values.

	Baseline (normal Krebs)	P&C	*P*-value	DAP5	*P*-value
A - 3 weeks after IOP elevation					
Age matched controls: Left (*n* = 9)	2 ± 0.06	2 ± 0.06	*P* = 0.272	2 ± 0.06	*P* = 0.594
Age matched controls: Right (*n* = 8)	1.8 ± 0.07	1.8 ± 0.08	*P* = 0.785	1.9 ± 0.07	*P* = 0.685
OHT: Left (input from unoperated eye) (*n* = 10)	2 ± 0.06	2 ± 0.06	*P* = 0.081	2 ± 0.05	*P* = 0.168
OHT: Right (input from operated eye) (*n* = 10)	1.8 ± 0.07	1.8 ± 0.05	*P* = 0.193	1.8 ± 0.05	*P* = 0.678
B - 16 weeks after IOP elevation					
Age matched controls: Left (*n* = 17)	2.1 ± 0.07	2.1 ± 0.07	*P* = 0.826	2.1 ± 0.07	*P* = 0.718
Age matched controls: Right (*n* = 14)	1.9 ± 0.08	1.9 ± 0.08	*P*= 1.000	1.9 ± 0.07	*P* = 0.336
Left (input from unoperated eye) (*n* = 10)	2 ± 0.09	2 ± 0.09	*P* = 0.443	2 ± 0.09	*P* = 0.591
Right (input from operated eye) (*n* = 10)	1.8 ± 0.07	1.8 ± 0.07	*P* = 0.853	1.8 ± 0.07	*P* = 0.279
C - 32 weeks after IOP elevation					
Age matched controls: Left (*n* = 15)	2 ± 0.05	2.1 ± 0.06	*P* = 0.843	2.1 ± 0.06	*P* = 0.765
Age matched controls: Right (*n* = 13)	1.8 ± 0.03	1.7 ± 0.04	*P* = 0.165	1.7 ± 0.03	*P* = 0.673
Left (input from unoperated eye) (*n* = 12)	1.9 ± 0.07	1.9 ± 0.06	*P* = 0.845	1.9 ± 0.09	*P* = 0.909
Right (input from operated eye) (*n* = 14)	1.8 ± 0.06	1.8 ± 0.07	*P* = 0.212	1.8 ± 0.08	*P* = 0.752

**Table 3:** Contribution of the GABA receptors to the fEPSP and contribution of the NMDA receptors to the excitatory fEPSP in age matched unoperated controls and OHT model animals. Values are average (%) ± SEM of *n* values.

	Contribution of GABA receptors to the fEPSP (%)	Contribution of NMDA receptors to the excitatory fEPSP (%)
A - 3 weeks after IOP elevation		
Age matched controls: Left (*n* = 9)	85.4 ± 1.4	62.7 ± 2.5
Age matched controls: Right (*n* = 8)	82 ± 1.2	65.5 ± 1.6
*P* value left vs. right	*P* = 0.191	*P* = 0.887
OHT: Left (input from unoperated eye) (*n* = 10)	86 ± 1.2	62 ± 2.7
OHT: Right (input from operated eye) (*n* = 10)	84 ± 0.7	64 ± 3.2
*P* value OHT left vs. right	*P* = 0.634	*P* = 0.947
B - 16 weeks after IOP elevation		
Age matched controls: Left (*n* = 17)	82.6 ± 1.7	53.4 ± 2.1
Age matched controls: Right (*n* = 14)	81.5 ± 2.9	55.5 ± 2.1
*P* value left vs. right	*P* = 0.981	*P* = 0.885
OHT: Left (input from unoperated eye) (*n* = 10)	85 ± 2.4	60 ± 2.4
OHT: Right (input from operated eye) (*n* = 10)	85 ± 1.1	62 ± 2.2
*P* value OHT left vs. right	*P* = 1.000	*P* = 0.971
C - 32 weeks after IOP elevation		
Age matched controls: Left (*n* = 15)	84.4 ± 1.1	57 ± 1.7
Age matched controls: Right (*n* = 13)	85.8 ± 1.3	62.9 ± 2.6
*P* value left vs. right	*P* = 0.933	*P* = 0.364
OHT: Left (input from unoperated eye) (*n* = 12)	83 ± 1.5	53 ± 2.9
OHT: Right (input from operated eye) (*n* = 14)	80 ± 2.4	67 ± 3
*P* value OHT left vs. right	*P* = 0.661	*P* = 0.001

**Table 4:** The effect of GABA and NMDA receptor antagonists on the duration of the fEPSP in age matched unoperated controls. Values are average (ms) ± SEM of *n* values.

	Baseline (normal Krebs)	P&C	*P*-value	DAP5	*P*-value
A - 10 weeks old (Equivalent 3 weeks after IOP elevation)					
Left (*n* = 9)	4.1 ± 0.19	96 ± 7.9	*P* = 0.000	33 ± 3.4	*P* = 0.000
Right (*n* = 8)	3.7 ± 0.11	90 ± 3.1	*P* = 0.000	26 ± 1.9	*P* = 0.000
B - 23 weeks old (Equivalent 16 weeks after IOP elevation)					
Left (*n* = 17)	4.3 ± 0.17	85 ± 6.0	*P* = 0.000	34 ± 2.5	*P* = 0.000
Right (*n* = 14)	4.1 ± 0.13	84 ± 6.3	*P* = 0.000	30 ± 2.3	*P* = 0.000
C - 39 weeks old (Equivalent 32 weeks after IOP elevation)					
Left (*n* = 15)	4.2 ± 0.24	75 ± 6.7	*P* = 0.000	26 ± 2.2	*P* = 0.000
Right (*n* = 13)	3.7 ± 0.17	72 ± 5.0	*P* = 0.000	21 ± 2.1	*P* = 0.000

There were no significant differences between the Left and Right slices during each condition (baseline, P&C, or DAP5) at each time point (*P* > 0.05).

**Table 5:** The effect of GABA and NMDA receptor antagonists on the duration of the fEPSP in OHT model animals. Values are average (ms) ± SEM of *n* values.

	Baseline (normal Krebs)	P&C	*P*-value	DAP5	*P*-value
A- 3 weeks after IOP elevation					
Left (input from unoperated eye) (*n* = 10)	4.3 ± 0.16	77 ± 3.7	*P* = 0.000	23 ± 1.6	*P* = 0.000
Right (input from operated eye) (*n* = 10)	4.1 ± 0.15	81 ± 4.1	*P* = 0.000	25 ± 2.2	*P* = 0.000
B - 16 weeks after IOP elevation					
Left (input from unoperated eye) (*n* = 10)	4.2 ± 0.22	73 ± 4.3	*P* = 0.000	23 ± 2.8	*P* = 0.000
Right (input from operated eye) (*n* = 10)	3.9 ± 0.13	79 ± 6.6	*P* = 0.000	26 ± 2.9	*P* = 0.000
C - 32 weeks after IOP elevation					
Left (input from unoperated eye) (*n* = 12)	4.2 ± 0.17	66 ± 5.5	*P* = 0.000	24 ± 2	*P* = 0.000
Right (input from operated eye) (*n* = 12)	3.9 ± 0.12	60 ± 3.1	*P* = 0.000	19 ± 0.96	*P* = 0.000

There were no significant differences between the Left and Right slices during each condition (baseline, P&C, or DAP5) at each time point (*P* > 0.05).

## References

[bib1] Arakawa T., Okada Y. (1988). Excitatory and inhibitory action of GABA on synaptic transmission in slices of guinea pig superior colliculus. Eur. J. Pharmacol..

[bib2] Binns K.E., Salt T.E. (1994). Excitatory amino acid receptors participate in synaptic transmission of visual responses in the superficial layers of the cat superior colliculus. Eur. J. Neurosci..

[bib3] Binns K.E., Salt T.E. (1997). Different roles for GABAA and GABAB receptors in visual processing in the rat superior colliculus. J. Physiol..

[bib4] Binns K.E., Salt T.E. (1998). Developmental changes in NMDA receptor-mediated visual activity in the rat superior colliculus, and the effect of dark rearing. Exp. Brain Res..

[bib5] Boller M., Schmidt M. (2001). Postnatal maturation of GABA(A) and GABA(C) receptor function in the mammalian superior colliculus. Eur. J. Neurosci..

[bib6] Boller M., Schmidt M. (2003). GABAC receptors in the rat superior colliculus and pretectum participate in synaptic neurotransmission. J. Neurophysiol..

[bib7] Bormann J. (2000). The 'ABC' of GABA receptors. Trends Pharmacol. Sci..

[bib8] Boue-Grabot E., Roudbaraki M., Bascles L., Tramu G., Bloch B., Garret M. (1998). Expression of GABA receptor rho subunits in rat brain. J. Neurochem..

[bib9] Bowery N.G., Hudson A.L., Price G.W. (1987). GABAA and GABAB receptor site distribution in the rat central nervous system. Neuroscience.

[bib10] Chalmers D.T., McCulloch J. (1991). Alterations in neurotransmitter receptors and glucose use after unilateral orbital enucleation. Brain Res..

[bib11] Chalmers D.T., McCulloch J. (1991). Selective alterations in glutamate receptor subtypes after unilateral orbital enucleation. Brain Res..

[bib12] Chu D.C., Albin R.L., Young A.B., Penney J.B. (1990). Distribution and kinetics of GABAB binding sites in rat central nervous system: a quantitative autoradiographic study. Neuroscience.

[bib13] Cirone J., Pothecary C.A., Turner J.P., Salt T.E. (2002). Group I metabotropic glutamate receptors (mGluRs) modulate visual responses in the superficial superior colliculus of the rat. J. Physiol..

[bib14] Citri A., Malenka R.C. (2008). Synaptic plasticity: multiple forms, functions, and mechanisms. Neuropsychopharmacology.

[bib15] Clark S.E., Garret M., Platt B. (2001). Postnatal alterations of GABA receptor profiles in the rat superior colliculus. Neuroscience.

[bib16] Cohen S., Greenberg M.E. (2008). Communication between the synapse and the nucleus in neuronal development, plasticity, and disease. Annu. Rev. Cell Dev. Biol..

[bib17] Collingridge G.L., Bliss T.V. (1995). Memories of NMDA receptors and LTP. Trends Neurosci..

[bib18] Constantine-Paton M. (2000). The plastic brain. Neurobiol. Dis..

[bib19] Cordeiro M.F., Guo L., Luong V., Harding G., Wang W., Jones H.E., Moss S.E., Sillito A.M., Fitzke F.W. (2004). Real-time imaging of single nerve cell apoptosis in retinal neurodegeneration. Proc. Natl. Acad. Sci. U. S. A..

[bib20] Dreher B., Sefton A.J., Ni S.Y., Nisbett G. (1985). The morphology, number, distribution and central projections of Class I retinal ganglion cells in albino and hooded rats. Brain Behav. Evol..

[bib21] Duncan R.O., Sample P.A., Weinreb R.N., Bowd C., Zangwill L.M. (2007). Retinotopic organization of primary visual cortex in glaucoma: a method for comparing cortical function with damage to the optic disk. Invest. Ophthalmol. Vis. Sci..

[bib22] Duncan R.O., Sample P.A., Weinreb R.N., Bowd C., Zangwill L.M. (2007). Retinotopic organization of primary visual cortex in glaucoma: Comparing fMRI measurements of cortical function with visual field loss. Prog. Retin. Eye Res..

[bib23] Guo L., Moss S.E., Alexander R.A., Ali R.R., Fitzke F.W., Cordeiro M.F. (2005). Retinal ganglion cell apoptosis in glaucoma is related to intraocular pressure and IOP-induced effects on extracellular matrix. Invest. Ophthalmol. Vis. Sci..

[bib24] Guo L., Tsatourian V., Luong V., Podoleanu A.G., Jackson D.A., Fitzke F.W., Cordeiro M.F. (2005). En face optical coherence tomography: a new method to analyse structural changes of the optic nerve head in rat glaucoma. Br. J. Ophthalmol..

[bib25] Guo L., Salt T.E., Maass A., Luong V., Moss S.E., Fitzke F.W., Cordeiro M.F. (2006). Assessment of neuroprotective effects of glutamate modulation on glaucoma-related retinal ganglion cell apoptosis in vivo. Invest. Ophthalmol. Vis. Sci..

[bib26] Guo L., Salt T.E., Luong V., Wood N., Cheung W., Maass A., Ferrari G., Russo-Marie F., Sillito A.M., Cheetham M.E., Moss S.E., Fitzke F.W., Cordeiro M.F. (2007). Targeting amyloid-beta in glaucoma treatment. Proc. Natl. Acad. Sci. U. S. A..

[bib27] Gupta N., Ly T., Zhang Q., Kaufman P.L., Weinreb R.N., Yucel Y.H. (2007). Chronic ocular hypertension induces dendrite pathology in the lateral geniculate nucleus of the brain. Exp. Eye Res..

[bib28] Hirai H., Tomita H., Okada Y. (1993). Inhibitory effect of GABA (gamma-aminobutyric acid) on the induction of long-term potentiation in guinea pig superior colliculus slices. Neurosci. Lett..

[bib29] Houser C.R., Lee M., Vaughn J.E. (1983). Immunocytochemical localization of glutamic acid decarboxylase in normal and deafferented superior colliculus: evidence for reorganization of gamma-aminobutyric acid synapses. J. Neurosci..

[bib30] Imamura K., Onoe H., Shimazawa M., Nozaki S., Wada Y., Kato K., Nakajima H., Mizuma H., Onoe K., Taniguchi T., Sasaoka M., Hara H., Tanaka S., Araie M., Watanabe Y. (2009). Molecular imaging reveals unique degenerative changes in experimental glaucoma. NeuroReport.

[bib31] Jeon C.J., Gurski M.R., Mize R.R. (1997). Glutamate containing neurons in the cat superior colliculus revealed by immunocytochemistry. Vis. Neurosci..

[bib32] King W.M., Sarup V., Sauve Y., Moreland C.M., Carpenter D.O., Sharma S.C. (2006). Expansion of visual receptive fields in experimental glaucoma. Vis. Neurosci..

[bib33] Kiyosawa M., Bosley T.M., Kushner M., Jamieson D., Alavi A., Savino P.J., Sergott R.C., Reivich M. (1989). Positron emission tomography to study the effect of eye closure and optic nerve damage on human cerebral glucose metabolism. Am. J. Ophthalmol..

[bib34] Lacey C.J., Pothecary C.A., Salt T.E. (2005). Modulation of retino-collicular transmission by Group III metabotropic glutamate receptors at different ages during development. Neuropharmacology.

[bib35] Liu M., Georgiou A., Guo L., Salt T.E., Cordeiro M.F. (2008). Dendritic pathology in the superior colliculus in a rat model of experimental glaucoma. Invest. Ophthalmol. Vis. Sci..

[bib36] Lund R.D., Lund J.S. (1971). Synaptic adjustment after deafferentation of the superior colliculus of the rat. Science.

[bib37] Malenka R.C., Nicoll R.A. (1993). NMDA-receptor-dependent synaptic plasticity: multiple forms and mechanisms. Trends Neurosci..

[bib38] Miki A., Nakajima T., Takagi M., Shirakashi M., Abe H. (1996). Detection of visual dysfunction in optic atrophy by functional magnetic resonance imaging during monocular visual stimulation. Am. J. Ophthalmol..

[bib39] Mize R.R. (1988). Immunocytochemical localization of gamma-aminobutyric acid (GABA) in the cat superior colliculus. J. Comp. Neurol..

[bib40] Mize R.R., Butler G.D. (1996). Postembedding immunocytochemistry demonstrates directly that both retinal and cortical terminals in the cat superior colliculus are glutamate immunoreactive. J. Comp. Neurol..

[bib41] Mize R.R., Salt T.E. (2004). Contribution of GABAergic inhibition to synaptic responses and LTD early in postnatal development in the rat superior colliculus. Eur. J. Neurosci..

[bib42] Morrison J.C. (2005). Elevated intraocular pressure and optic nerve injury models in the rat. J. Glaucoma.

[bib43] Morrison J.C., Moore C.G., Deppmeier L.M., Gold B.G., Meshul C.K., Johnson E.C. (1997). A rat model of chronic pressure-induced optic nerve damage. Exp. Eye Res..

[bib44] Pang I.H., Clark A.F. (2007). Rodent models for glaucoma retinopathy and optic neuropathy. J. Glaucoma.

[bib45] Pasternack M., Boller M., Pau B., Schmidt M. (1999). GABA(A) and GABA(C) receptors have contrasting effects on excitability in superior colliculus. J. Neurophysiol..

[bib46] Platt B., Withington D.J. (1997). Response habituation in the superficial layers of the guinea-pig superior colliculus in vitro. Neurosci. Lett..

[bib47] Platt B., Withington D.J. (1998). GABA-induced long-term potentiation in the guinea-pig superior colliculus. Neuropharmacology.

[bib48] Pothecary C.A., Jane D.E., Salt T.E. (2002). Reduction of excitatory transmission in the retino-collicular pathway via selective activation of mGlu8 receptors by DCPG. Neuropharmacology.

[bib49] Pothecary C.A., Thompson H., Salt T.E. (2005). Changes in glutamate receptor function in synaptic input to the superficial superior colliculus (SSC) with aging and in retinal degeneration in the Royal College of Surgeons (RCS) rat. Neurobiol. Aging.

[bib50] Rao V.R., Finkbeiner S. (2007). NMDA and AMPA receptors: old channels, new tricks. Trends Neurosci..

[bib51] Resnikoff S., Pascolini D., Etya'ale D., Kocur I., Pararajasegaram R., Pokharel G.P., Mariotti S.P. (2004). Global data on visual impairment in the year 2002. Bull. World Health Organ..

[bib52] Roberts W.A., Eaton S.A., Salt T.E. (1991). Excitatory amino acid receptors mediate synaptic responses to visual stimuli in superior colliculus neurones of the rat. Neurosci. Lett..

[bib53] Schmidt M., Boller M., Ozen G., Hall W.C. (2001). Disinhibition in rat superior colliculus mediated by GABAc receptors. J. Neurosci..

[bib54] Segu L., Abdelkefi J., Dusticier G., Lanoir J. (1986). High-affinity serotonin binding sites: autoradiographic evidence for their location on retinal afferents in the rat superior colliculus. Brain Res..

[bib55] Shi J., Aamodt S.M., Constantine-Paton M. (1997). Temporal correlations between functional and molecular changes in NMDA receptors and GABA neurotransmission in the superior colliculus. J. Neurosci..

[bib56] Simon D.K., Prusky G.T., O'Leary D.D., Constantine-Paton M. (1992). N-methyl-D-aspartate receptor antagonists disrupt the formation of a mammalian neural map. Proc. Natl. Acad. Sci. U. S. A..

[bib57] Smith E.L., Chino Y.M., Harwerth R.S., Ridder W.H., Crawford M.L., DeSantis L. (1993). Retinal inputs to the monkey's lateral geniculate nucleus in experimental glaucoma. Clin. Vision Sci..

[bib58] Sugiyama T., Utsunomiya K., Ota H., Ogura Y., Narabayashi I., Ikeda T. (2006). Comparative study of cerebral blood flow in patients with normal-tension glaucoma and control subjects. Am. J. Ophthalmol..

[bib59] Turner J.P., Sauve Y., Varela-Rodriguez C., Lund R.D., Salt T.E. (2005). Recruitment of local excitatory circuits in the superior colliculus following deafferentation and the regeneration of retinocollicular inputs. Eur. J. Neurosci..

[bib60] Wegelius K., Pasternack M., Hiltunen J.O., Rivera C., Kaila K., Saarma M., Reeben M. (1998). Distribution of GABA receptor rho subunit transcripts in the rat brain. Eur. J. Neurosci..

[bib61] White A.M., Platt B. (2000). Age- and species-dependent maturation of synaptic transmission in the superficial superior colliculus. Eur. J. Neurosci..

[bib62] White A.M., Platt B. (2001). Ionic mechanisms of GABA-induced long-term potentiation in the rat superior colliculus. Exp. Brain Res..

[bib63] Yucel Y., Darabie A., Wang S., Kaufman P.L., Gupta N. (2006). Glutamate NMDA receptor 1 subunit expression is increased in the lateral geniculate nucleus of experimental glaucoma. Invest. Ophthalmol. Vis. Sci..

